# Acetylcholinesterase of the sand fly, *Phlebotomus papatasi* (Scopoli): cDNA sequence, baculovirus expression, and biochemical properties

**DOI:** 10.1186/1756-3305-6-31

**Published:** 2013-02-04

**Authors:** Kevin B Temeyer, Danett K Brake, Alexander P Tuckow, Andrew Y Li, Adalberto A Pérez deLeón

**Affiliations:** 1 Knipling-Bushland U.S. Livestock Insects Research Laboratory, Agricultural Research Service, U. S. Department of Agriculture, 2700 Fredericksburg Road, Kerrville, TX, 78028-9184, USA; 2 Present address: Medical Service Corps, U.S. Navy, Wound Infections Dept., Naval Medical Research Center, 503 Robert Grant Ave, Silver Spring, MD, 20910-7500, USA

**Keywords:** Sand fly, Acetylcholinesterase, *P. papatasi*, cDNA, AChE, JQ922267, AFP20868

## Abstract

**Background:**

Millions of people and domestic animals around the world are affected by leishmaniasis, a disease caused by various species of flagellated protozoans in the genus *Leishmania* that are transmitted by several sand fly species. Insecticides are widely used for sand fly population control to try to reduce or interrupt *Leishmania* transmission. Zoonotic cutaneous leishmaniasis caused by *L*. *major* is vectored mainly by *Phlebotomus papatasi* (Scopoli) in Asia and Africa. Organophosphates comprise a class of insecticides used for sand fly control, which act through the inhibition of acetylcholinesterase (AChE) in the central nervous system. Point mutations producing an altered, insensitive AChE are a major mechanism of organophosphate resistance in insects and preliminary evidence for organophosphate-insensitive AChE has been reported in sand flies. This report describes the identification of complementary DNA for an AChE in *P*. *papatasi* and the biochemical characterization of recombinant *P*. *papatasi* AChE.

**Methods:**

A *P. papatasi* Israeli strain laboratory colony was utilized to prepare total RNA utilized as template for RT-PCR amplification and sequencing of cDNA encoding acetylcholinesterase 1 using gene specific primers and 3’-5’-RACE. The cDNA was cloned into pBlueBac4.5/V5-His TOPO, and expressed by baculovirus in Sf21 insect cells in serum-free medium. Recombinant *P. papatasi* acetylcholinesterase was biochemically characterized using a modified Ellman’s assay in microplates.

**Results:**

A 2309 nucleotide sequence of *PpAChE1* cDNA [GenBank: JQ922267] of *P. papatasi* from a laboratory colony susceptible to insecticides is reported with 73-83% nucleotide identity to acetylcholinesterase mRNA sequences of *Culex tritaeniorhynchus* and *Lutzomyia longipalpis*, respectively. The *P. papatasi* cDNA ORF encoded a 710-amino acid protein [GenBank: AFP20868] exhibiting 85% amino acid identity with acetylcholinesterases of *Cx. pipiens*, *Aedes aegypti*, and 92% amino acid identity for *L. longipalpis*. Recombinant *P. papatasi* AChE1 was expressed in the baculovirus system and characterized as an insect acetylcholinesterase with substrate preference for acetylthiocholine and inhibition at high substrate concentration. Enzyme activity was strongly inhibited by eserine, BW284c51, malaoxon, and paraoxon, and was insensitive to the butyrylcholinesterase inhibitors ethopropazine and iso-OMPA.

**Conclusions:**

Results presented here enable the screening and identification of *PpAChE* mutations resulting in the genotype for insensitive PpAChE. Use of the recombinant *P. papatasi* AChE1 will facilitate rapid in vitro screening to identify novel PpAChE inhibitors, and comparative studies on biochemical kinetics of inhibition.

## Background

Over 350 million people are at risk of suffering from leishmaniasis and over 2 million new cases are reported each year, which makes it one of the most neglected diseases globally [1,2]. The sand-fly *Phlebotomus papatasi* (Scopoli) is the vector of the flagellate protozoan *Leishmania major* that causes zoonotic cutaneous leishmaniasis [3-5] in Asia and Africa [1,6,7]. Various species of burrowing rodents are reservoir hosts of *L. major* and sand flies are closely associated with the rodent burrows making vector control by direct insecticidal application difficult to achieve [6,8-10]. Prevention of *Leishmania* spp. transmission to humans is largely reliant on vector control to avoid insect bites by use of screens, insecticide impregnated bed nets or curtains, establishment of barrier zones by insecticide applications, or environmental modification [2,9,11,12]. Use of synthetic sand fly pheromone as an attractant was reported to improve effectiveness of pesticide application for control of the new world sand fly *Lutzomyia longipalpis* in chicken sheds [13]. Attempts to control *Phlebotomus papatasi* by various types of insecticide application has had questionable effectiveness, particularly in the harsh environments reported during combat operations by the U.S. military in Iraq [14,15], although there was no evidence of significant insecticide resistance.

Insecticide resistance is an increasing problem in control of insect vectors of disease and there have been reports of possible resistance in sand fly populations to various insecticides [2,11,16]. Pesticide effectiveness for knockdown of Phlebotomine sand flies in Morocco was found to be variable for different fly populations, reflective of past history of high pesticide use in some areas for malaria control, although all of the sand flies tested as susceptible to the pesticides by bioassay, suggesting possible genetic changes that might lead to development of resistance [17]. Preliminary evidence for an altered acetylcholinesterase (E.C. 3.1.1.7, AChE) was reported in the sand fly *Phlebotomus argentipes* from Sri Lanka [18], and from *Phlebotomus papatasi* in Khartoum State, Sudan [19], each possibly the result of widespread use of insecticides in the Anti-Malarial Campaign. Coutinho-Abreu et al. [20] reported that the AChE gene of *Lutzomyia longipalpis* (Lutz & Neiva) appears to be structurally similar to other insect species in which point mutations result in an altered enzyme insensitive to organophosphate or carbamate inhibitors. Organophosphate pesticides function as quasi-irreversible inhibitors of AChE, resulting in failure of the central nervous system and death of the insect. Point mutations within the sequence encoding AChE resulting in production of an altered, insensitive enzyme have been reported to be a major mechanism of organophosphate resistance in insects [21-26]. Determination of the nucleotide sequence encoding AChE of *P. papatasi* should enable rapid identification of mutations potentially associated with resistance, and the development of rapid molecular tests to screen *P. papatasi* populations for the presence of specific mutations. Confirmation of AChE mutations resulting in production of an insensitive AChE is expected to facilitate the development of rapid molecular tests for pesticide resistance in sand flies. This report describes the identification of complementary DNA for AChE1 in *P. papatasi* and the biochemical characterization of recombinant *P. papatasi* AChE1 produced using a baculovirus expression system.

## Methods

### Sand flies

Sand flies used in this study were from a *P. papatasi* colony maintained at the USDA-ARS, Knipling-Bushland U.S. Livestock Insects Research Laboratory in Kerrville, Texas. The colony was established using pupae from an Israeli strain of *P. papatasi* maintained for 30 years and never exposed to pesticides at the Division of Entomology, Walter Reed Army Institute of Research (Silver Spring, Maryland). The colony is therefore considered to be generally susceptible to insecticides and baseline data on pesticide susceptibility by bioassay of colony sand flies is being collected for publication (A. Li, personal communication). Larvae were fed with a sand fly larval diet, a mixture of fermented rabbit feces and rabbit food [27]. Both larvae and adult flies were maintained at 26±2°C and a relative humidity of 85±2%. Males were fed with 30% sucrose water and females were fed with defibrinated cattle blood using an in vitro membrane feeding system. Larvae and newly emerged unfed adult male flies were collected and quick-frozen by immersion in liquid nitrogen, and stored at −70°C until use.

### RNA preparation

Total RNA was prepared from frozen whole larvae or unfed adults by grinding in a glass tissue homogenizer containing Tri-Reagent (Sigma Chemical, St. Louis, MO) or by use of a MagMAX™-96 RNA Isolation Kit (Life Technologies, Carlsbad, CA) according to the manufacturers’ instructions.

### Oligonucleotide synthesis

Synthetic oligodeoxynucleotides were purchased from Sigma-Genosys (The Woodlands, TX).

### cDNA synthesis

Complementary DNA (cDNA) was synthesized by reverse transcription from template RNA using the SuperScript™ Choice System for cDNA Synthesis (Life Technologies) according to the manufacturer’s instructions. Oligodeoxynucleotide primers were oligo(dT_18_V) or gene-specific primers based on known sequence.

### Polymerase chain reaction (PCR)

We utilized automatic hot-start PCR using AmpliTaq Gold® DNA polymerase (Applied Biosystems, Foster City, CA) or Advantage® 2 HF DNA Polymerase (BD Biosciences Clontech, Mountain View, CA) according to the manufacturers’ instructions.

### Agarose gel electrophoresis

DNA preparations generated from PCR, RT-PCR, or bacterial plasmids were analyzed by agarose gel electrophoresis in tris-borate-EDTA buffer by standard techniques [28]. DNA bands were visualized by staining with GelStar® Nucleic Acid Gel Stain (Cambrex, Rockland, MD) and photographed on a Kodak GelLogic 440 Imaging system (Eastman Kodak Co., Rochester, NY) with uv illumination.

### Rapid Amplification of cDNA Ends (RACE)

5’- and 3’-RACE was performed using the SMARTer™ RACE cDNA Amplification Kit (BD Biosciences Clontech, Palo Alto, CA).

### DNA cloning

Double-stranded cDNA was inserted into bacterial plasmid DNA using the TOPO TA Cloning® Kit for Sequencing (Invitrogen, Carlsbad, CA), transformed into One Shot® TOP10 chemically competent *Escherichia coli* (Invitrogen) for amplification, screened for appropriate inserts by PCR, and sequenced by normal sequencing procedures (see below).

### DNA sequencing

For sequencing, PCR amplification reactions were scaled up to 50 μl and purified using ExoSAP-IT® (Affymetrix, Santa Clara, CA). The sequencing reaction using BigDye® terminator (Applied Biosystems) and precipitation of the products with ethanol were performed according to the manufacturer’s instructions. Sequencing products were analyzed on a PRISM 3130*xl* Genetic Analyzer (Applied Biosystems).

### Analysis of amino acid or nucleotide sequences

Sequence analysis to design oligonucleotide primers used for PCR or sequencing utilized OLIGO® Primer Analysis Software, Version 5.0 (1996, National Biosciences Inc, Plymouth, MN). Sequence chromatograms and sequence assembly utilized ChromasPro ver. 1.32 (Technelysium Pty Ltd., available at http://technelysium.com.au/?page_id=27). Clustal W (http://www.ebi.ac.uk/Tools/msa/clustalw2/) was used to perform multiple sequence alignments [29]. BLAST search of GenBank (http://blast.ncbi.nlm.nih.gov/Blast.cgi), [30] and on-line protein sequence analyses were done using ExPASy Proteomics tools.

### PpAChE expression construct

Double stranded cDNA containing the complete PpAChE1 coding sequence [GenBank: JQ922267] was obtained by PCR amplification of cDNA using PpAce18F-273U23 and PpAce18F-2561 L20 (Table 1) using an annealing temperature of 62.5°C and extension time of 3 min. at 72°C. The cDNA was cloned into pBlueBac4.5/V5-His TOPO® (Applied Biosystems/Life Technologies) and sequenced to verify proper construction.

**Table 1:** **Oligodeoxynucleotide primers for AChE of *P. papatasi***

**Oligonucleotide name**	**Position**^**a**^	**Nucleotide Sequence**^**b**^
PpAce18F-273U23	1-23 (U)	CAATAACGTGGTATCTCGCATAA
PpAChE458U17	186-202 (U)	TTGGCGGAGGGTCGTCA
PpAChE552U22	280-302 (U)	TCTTAGGCGAATCAACATTAGA
PpAChE550L23	300-278 (L)	CTAATGTTGATTCGCCTAAGACT
PpAChE5R-693 L20	497-378 (L)	CGCTCCAGTGTGCCCAATTC
PpAChE20-828U23	827-849 (U)	CTTCGGTGGTGGATTCTACTCAG
PpAChEp13-435 L21	975-955 (L)	CAGGGGCATCAGGAGTACCAA
PpAChEp13-719U18	1221-1239 (U)	CTAGCCGAAGCCGTGGAG
PpAChE7Jan-590 L22	1226-1205 (L)	GGCTAGGCGAAGGGTTCTATTG
PpAChE7J-887U23	1502-1524 (U)	AGAGGAGGGCATAACTGTAACAC
PpAChE7J-947 L22	1582-1562 (L)	CGCACGGCACCATTGACATAG
PpAChE7J-1100U22	1715-1736 (U)	TGAGGAGGGCAACAATGTCTAC
PpAce2L27	1749-1723 (L)	TGTAGAGATACATGTAGACATTGTTGC
PpAce22L18	1760-1743 (L)	GGTGCGATGGGTGTAGAG
PpAce18F-2561 L20	2309-2289 (L)	GAGTAAATCGCGTTACTTCA

^a^Position (based on PpAChE sequence numbering, Figure 1).

^b^Sequence is 5’**s**3’, upper (U) or lower (L) strand.

### Baculovirus expression of recombinant PpAChE

*PpAChE1* clones were constructed, sequenced, and expressed as baculovirus expression clones in Sf21 insect cells in sf900 III serum-free medium (Life Technologies/Gibco) as previously described [31,32]. Serum-free cell culture baculovirus lysates were collected and centrifuged to remove cell debris and supernatants were used for biochemical characterization of recombinant PpAChE1.

### Biochemical characterization of rPpAChE1

AChE activity of baculovirus culture supernatant was measured by a modified Ellman’s method as described previously [33] except that the reaction was monitored every minute for 30 min to establish initial velocity. For inhibition studies, rPpAChE was preincubated with an appropriate concentration of the inhibitor at 23°C for 10 min in 100 μl in the microtiter plate without substrate or DTNB [(5’.5’-dithiobis-(2-nitrobenzoic acid)] and AChE activity was monitored for 10 min after initiation of the reaction by addition of 100 μl substrate and DTNB. Cholinesterase inhibitors included the AChE-specific inhibitors eserine and BW284c51, butyrylcholinesterase-specific inhibitors ethopropazine and iso-OMPA, and oxidized organophosphates, paraoxon and malaoxon. All chemicals were of reagent grade obtained from Sigma Chemical Co. Data were analyzed and plotted using GraphPad Prism ver. 5.0 (GraphPad Prism, Inc., La Jolla, CA).

## Results

Beginning with a presumptive acetylcholinesterase partial sequence (111 nucleotides) of *Phlebotomus perniciosus* [GenBank: AJ865843], we constructed oligodeoxynucleotide primers for 5’-, and 3’-RACE to obtain the complete *PpAChE* cDNA sequence [GenBank: JQ922267] (Additional file 1). Both strands were sequenced for the entire presumptive coding sequence of the cDNA at least three times. Oligodeoxynucleotide primers used for sequencing or 3’-/5’-RACE are listed in Table 1. The sequence obtained for the presumptive *PpAChE1* cDNA of *P.  papatasi* was comprised of 2309 nucleotides organized as a 71-nucleotide 5’-untranslated region, an open reading frame (ORF) of 2130 nucleotides, and a 105-nucleotide 3’-untranslated region. BLAST homology search [30] of GenBank (11,299,630 sequences) found significant nucleotide identity (73-83%) to the AChE mRNA sequences of a mosquito, *Culex tritaeniorhynchus* [GenBank: AB122152] and for the new world sand fly *L. longipalpis* (partial codes, 1188 nucleotides, [GenBank: DQ898276]) respectively, as well as to other arthropod AChEs.

**Figure 1:**
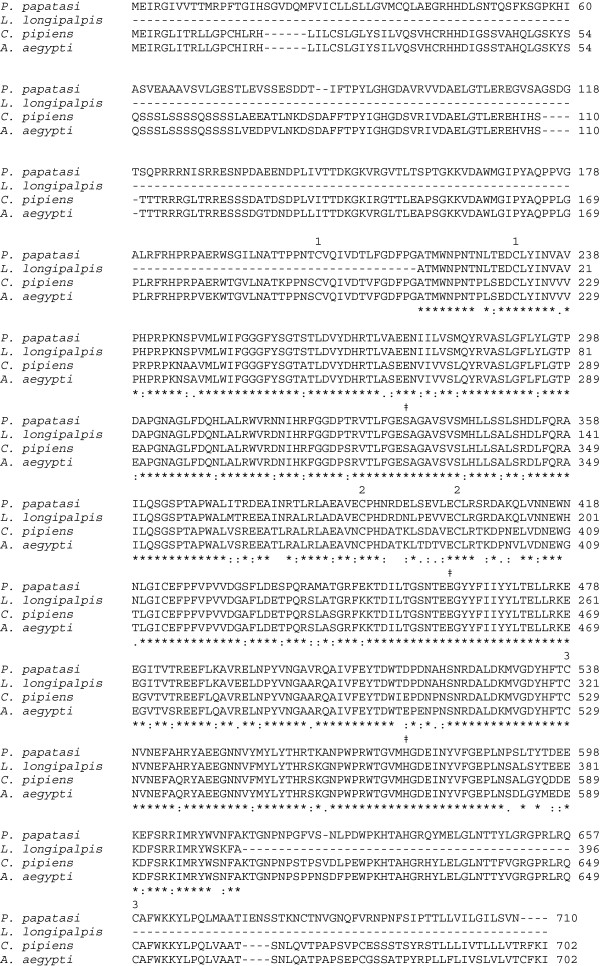
**Clustal W 2.1 multiple sequence alignment of AChE protein sequences for *Phlebotomus papatasi *[GenBank: JQ922267], *Lutzomyia longipalpis *[GenBank: ABI74669], *Culex pipiens *AChE [GenBank: Q86GC8], and *Aedes aegypti *[GenBank: XP_001656977].** The consensus line below the aligned sequences indicates positions of conserved amino acid identity (*) or similarity (: or .). Positions of the 3 disulfide bond linkages are indicated by numbers above participating cysteine pairs. The members of the catalytic triad (S, E, H) which make up the catalytic site are indicated by (‡) above the participating amino acid.

The *P. papatasi* AChE cDNA ORF encodes a 710-amino acid protein [GenBank: AFP20868]; 79451 MW, 5.77 pI, http://web.expasy.org/compute_pi/) exhibiting 85% amino acid identity with AChEs of mosquitoes, *Culex pipiens* [GenBank: Q86GC8], *Aedes aegypti* [GenBank: XP_001656977], and 92% amino acid identity with the new world sand fly, *L. longipalpis* AChE (partial codes, 396 amino acids, [GenBank: ABI74669]. Comparison of the amino acid sequence of PpAChE1 with the AChE protein of *L. longipalpis* (partial codes) and the AChEs of *Cx. pipiens* and *Ae. aegypti* by Clustal W multiple sequence alignment is shown in Figure 1. As seen in Figure 1, there was very high amino acid identity throughout the partial sequence of *L. longipalpis* AChE as well as substantial identity with the two mosquito AChEs. Presumptive disulfide bonds, members of the catalytic triad, and residues lining the catalytic gorge [34,35] are indicated in the Clustal W multiple sequence alignment with AChEs of *Torpedo californica* [GenBank: 1EA5_A] and *Drosophila melanogaster* [GenBank: 1QO9_A] (Additional file 2).

Recombinant PpAChE1 was expressed in the baculovirus system and used for biochemical characterization. Biochemical properties (substrate *K*_m_ and relative sensitivities to inhibitors) obtained for rPpAChE1 are presented in Table 2. The enzyme was strongly inhibited by eserine, paraoxon, malaoxon, and the acetylcholinesterase inhibitor Bw284c51 and exhibited much lower sensitivity to the butyrylcholinesterase inhibitors iso-OMPA and ethopropazine. In addition, rPpAChE1 hydrolyzed 0.1 mM acetylthiocholine (AcSCh) 17-fold faster than 0.1 mM butyrylthiocholine (BuSCh), demonstrating substrate preference for AcSCh over BuSCh and was inhibited at substrate concentrations above 5 mM.

**Table 2:** Biochemical properties of rPpAChE

**Property**^**a**^	**PpAChE**^**b**^
*K*_m_ AcSCh (μM)	37.9 ± 1.6
IC_50_ Paraoxon (10^-7^ M)	2.3 (1.9-2.7)
IC_50_ Malaoxon (10^-8^ M)	2.7 (1.7-4.5)
IC_50_ Eserine (10^-10^ M)	7.2 (5.0-10.5)
IC_50_ BW284c51 (10^-8^ M)	7.1 (5.9-8.4)
IC_50_ Ethopropazine (10^-6^ M)	4.4 (4.0-4.9)
IC_50_ Iso-OMPA	NC^c^

^a^AcSCh = acetylthiocholine; IC_50_ = concentration of inhibitor producing 50% inhibition of enzyme activity during 10 min preincubation.

^b^Data are presented as mean ± SE or (95% confidence interval).

^c^ NC = not calculated (no inhibition detected at 10^-3^ M).

## Discussion

Biochemical properties of rPpAChE1 presented in this report are consistent with classification as an insect acetylcholinesterase as defined by Toutant [36]. There is substrate preference for acetylthiocholine over butyrylthiocholine, significant inhibition by the AChE-specific inhibitors eserine and BW284c51 with relative insensitivity to inhibitors (ethopropazine and iso-OMPA) classified as specific for butyrylcholinesterase. The enzyme is strongly inhibited by paraoxon and malaoxon (oxidized forms of parathion and malathion) and exhibits a *K*_m_ for acetylthiocholine (37.9 μM) similar to values reported for recombinant acetylcholinesterases of *Ae. aegypti* (13.79 μM) [37], *Stomoxys calcitrans* (63.9 μM) [33], *Haematobia irritans* (31.3 μM) [38], or *Musca domestica* (150 μM) [39].

*P. papatasi* is a significant public health problem in the Middle East, Asia and Africa where it transmits *L*. *major* that causes zoonotic cutaneous leishmaniasis in humans [40]. Additionally, zoonotic cutaneous leishmaniasis can seriously affect the operational readiness of United States military personnel deployed in Iraq and Afghanistan [41]. Attempts by the U.S. military to control sand flies through the use of chemical pesticides were not highly successful, possibly due to extreme environmental conditions [14,15], and pesticide bioassays did not reveal evidence of insecticide resistance. Chemical pesticides have been used in several ways for insect control, including area-wide applications for malaria control in the same areas inhabited by *P. papatasi*. Recent reports of pesticide resistance in sand flies, possibly resulting from insensitive AChE [18,19], highlight the urgent need to characterize the mechanisms responsible for insecticide resistance and the relevance of efforts to develop rapid tests to identify resistant pest populations.

Point mutations in pest AChEs have been shown to generate altered enzymes with decreased inhibition by organophosphate [16,21,42-45]. The present report demonstrating very high amino acid sequence identity of the *P. papatasi* AChE to those of *Cx. pipiens* and *Ae. aegypti* strongly suggests that this PpAChE1 is the target site for organophosphate and will facilitate identification of *PpAChE1* mutations that produce insensitivity to organophosphate or carbamate pesticides.

In mosquito vectors, very high resistance to OPs and carbamates results from a single amino acid substitution of Serine for Glycine (codon GGC, *PpAChE1* pos 837, Gly→AGC, Ser) [46-50]. *Ae. aegypti* is reported to have the GGA codon at this position (as does our *PpAChE1*), and when converted to AGC, the *A. aegypti* enzyme has the identical high level resistance as *Anopheles*[49]. It has been suggested that GGA at this position is extremely unlikely to evolve to AGC, and that in nature, only Gly or Ser are found at this position, which indicates that no other amino acids are allowed in AChE1 at that position [49]. Our results suggest significant OP-insensitivity in AChE of *P. papatasi* would be unlikely to involve PpAChE1, unless nucleotide pos 839 exists in a polymorphic state (i.e., GGA/C) in nature. Alternatively, it is hypothesized that, if present, other AChEs in *P. papatasi* could provide the mechanism to develop target insensitivity as documented in *Ae*. *aegypti* where a high level of AChE insensitivity was due to substitutions at positions F455W (*Tc* pos Phe331) and Ile697 of AChE2 [51,52].

## Conclusions

Use of the recombinant *P. papatasi* AChE1 will facilitate rapid in vitro screening to identify novel PpAChE inhibitors, and comparative studies on biochemical kinetics of inhibition. Information on the identification and characterization of PpAChE1 presented here will facilitate the development of a rapid molecular assay for the GGC codon (pos. 839) in *P. papatasi* populations, and other molecular tools to screen for mutations giving rise to an organophosphate-insensitive PpAChE1. Molecular data on PpAChE1 could also be used in modeling studies to predict in vivo insecticidal activity for novel inhibitors as described by Naik et al. [53]. Availability of the recombinant PpAChE1 will enable the creation of mechanism-based screens to discover more effective inhibitors that may be developed to innovate safer vector control technologies. Novel synthetic carbamates have already been identified by screening using the recombinant PpAChE that present essentially equivalent inhibition of the target AChE with substantially improved target specificity resulting in significantly enhanced safety [Swale DR, Tong F, Temeyer KB, Li AY, Totrov MM, Carlier PR, unpublished].

### Endnotes

^1^This article reports the results of research only. Mention of a proprietary product does not constitute an endorsement by the USDA for its use.

^2^In conducting research described in this report, investigators adhered to the “Guide for the Care and Use of Laboratory Animals,” as promulgated by the Committee on Care and Use of Laboratory Animals of the Institute of Laboratory Animal Resources, National Research Council. A protocol describing routing procedures for animal care and use is on file with the Animal Care Committee at the research location.

^3^USDA is an equal opportunity provider and employer.

^4^Copyright statement: Copyright protection is not available for any work of the United States Government.

^5^Disclaimer: "The views expressed in this article are those of the authors and do not necessarily reflect the official policy or position of the Department of the Navy, Department of Defense, nor the U.S Government.”

## Abbreviations

(AChE): Acetylcholinesterase; (DTNB): Dithiobisnitrobenzoic acid; (AcSCh): Acetylthiocholine; (BuSCh): Butyrylthiocholine; (rPpAChE1): Recombinant *P. papatasi* AChE1.

## Competing interests

The authors declare that they have no competing interests.

## Authors’ contributions

KBT, AYL, and AAPdL conceived experiments to clone the PpAChE and biochemically characterize its recombinant form. KBT designed experiments and supervised laboratory tests to identify, clone, sequence, and express rPpAChE in the baculovirus system. DKB and APT conducted biochemical assays and data analysis of rPpAChE1. AYL and AAPdL obtained DWFP funding, maintained the sand fly colony, and provided sand flies for experimental use. All authors contributed to writing and revision of the manuscript and approved the final version.

## Supplementary Material

Additional file 1: Figure S1 Nucleotide and amino acid sequences are listed for *Phlebotomus papatasi* AChE for rPpAChE-16 expressed in the baculovirus system. Click here for file

Additional file 2: Figure S2 Clustal W 2.1 multiple sequence alignment of AChE protein sequences for *Torpedo californica* [GenBank: 1EA5_A], *Phlebotomus papatasi* (rPpAChE-16), and *Drosophila melanogaster* AChE [GenBank: 1QO9_A]. The consensus line below the aligned sequences indicates positions of conserved amino acid identity (*) or similarity (: or.). Positions of the 3 disulfide bond linkages are indicated by numbers above participating cysteine pairs. The members of the catalytic triad (S, E, H) which make up the catalytic site are indicated by (‡) above the participating amino acid. Positions lining the catalytic gorge are indicated by (▼) above the participating amino acid [32,33]. Click here for file
