# Supplementary material for: Acetylcholinesterase of the sand fly, Phlebotomus papatasi (Scopoli): cDNA sequence, baculovirus expression, and biochemical properties
Source: Parasit Vectors. 2013 Feb 4;6:31. doi: 10.1186/1756-3305-6-31 (PMC3598880; doi:10.1186/1756-3305-6-31)
Supplement: Additional file 1: Figure S1 — Nucleotide and amino acid sequences are listed for Phlebotomus papatasi AChE for rPpAChE-16 expressed in the baculovirus system. [file 1756-3305-6-31-S1.pdf]

Supplemental Fig. 1

>PpAChE1-16
CAATAACGTGGTATCTCGCATAATATGTTTCTACATGTGCATTTGAGGTGTTGACAAACGGTTTCTCCAGGATGGAG
ATTCGGGGCATTGTTGTCACCACAATGCGCCCCTTCACAGGCATCCACAGCGGTGTTGACCAAATGTTTGTAATATG
TCTCTTGAGTCTACTTGGAGTGATGTGCCAATTGGCGGAGGGTCGTCACCACGACCTAAGCAATACGCAATCATTTA
AGTCGGGGCCAAAGCACATTGCATCCGTGGAAGCGGCAGCGGTGTCAGTCTTAGGCGAATCAACATTAGAAGTATCA
AGCGAGAGTGATGATACAATTTTTACCCCCTATCTTGGCCATGGTGATGCAGTGAGAGTTGTGGATGCGGAATTGGG
CACACTGGAGCGTGAAGGTGTGTCTGCTGGGAGTGACGGCACATCTCAGCCACGACGTCGCAATATCAGCCGGCGCG
AATCAAATCCGGATGCTGAGGAAAACGATCCTCTGATTGTGACAACGGATAAGGGGAAGGTGAGAGGTGTTACATTA
ACATCTCCAACGGGTAAGAAGGTGGATGCGTGGATGGGGATTCCGTACGCACAGCCACCTGTTGGTGCACTGCGCTT
CCGACATCCACGACCGGCCGAACGGTGGTCCGGAATCCTCAATGCCACAACCCCGCCAAATACGTGTGTTCAGATTG
TCGATACGCTATTCGGCGACTTCCCGGGCGCCACTATGTGGAATCCTAACACTAACCTTACGGAGGATTGTCTCTAC
ATTAATGTCGCAGTACCACATCCACGTCCCAAAAACTCACCAGTTATGCTATGGATCTTCGGTGGT**GGA**TTCTACTC
AGGAACATCCACACTCGATGTGTACGATCACAGGACCCTTGTAGCGGAGGAAAACATCATTCTGGTATCTATGCAGT
ATCGTGTGGCTAGCCTGGGCTTCCTCTATCTTGGTACTCCTGATGCCCCTGGCAATGCGGGTCTTTTCGATCAACAT
CTCGCTCTGCGATGGGTACGAAATAACATTCATAGATTCGGCGGTGATCCTACAAGGGTTACACTGTTTGGTGAAAG
TGCCGGTGCTGTGTCGGTTTCCATGCATCTACTGTCGTCTTTATCACATGATCTCTTCCAACGTGCTATCCTTCAGA
GTGGTTCACCAACGGCGCCGTGGGCCCTCATTACGCGCGATGAAGCCATCAATAGAACCCTTCGCCTAGCCGAAGCC
GTGGAGTGCCCTCACAATCGAGATGAGCTATCTGAAGTGCTGGAATGCTTGCGTAGCAGGGATGCCAAACAACTGGT
TAACAATGAGTGGAACAATCTCGGAATATGTGAGTTTCCTTTTGTACCAGTTGTAGATGGATCCTTTCTTGATGAGT
CACCACAGAGAGCAATGGCAACTGGCCGTTTTGAGAAGACGGACATCCTCACGGGAAGTAATACTGAGGAGGGATAC
TACTTCATCATATACTATCTAACTGAATTGCTGAGAAAAGAAGAGGGCATAACTGTAACACGTGAAGAGTTCCTCAA
GGCTGTTCGAGAACTCAATCCCTATGTCAATGGTGCCGTGCGTCAAGCTATCGTATTTGAGTATACAGACTGGACTG
ATCCGGATAATGCTCATAGCAATCGAGATGCACTGGATAAAATGGTTGGTGACTATCATTTCACGTGCAATGTGAAT
GAATTTGCTCACAGATATGCTGAGGAGGGCAACAATGTCTACATGTATCTCTACACCCATCGCACCAAAGCAAATCC
ATGGCCACGCTGGACGGGAGTTATGCATGGGGATGAAATTAACTATGTATTTGGAGAGCCTCTCAATCCTTCACTGA
CTTATACGGACGAAGAGAAGGAATTCAGCCGGAGGATTATGAGATATTGGGTGAACTTTGCAAAGACCGGAAATCCC
AACCCTGGATTTGTATCAAACCTGCCCGATTGGCCAAAACATACTGCTCATGGAAGACAATACATGGAATTAGGGCT
CAATACGACTTACCTCGGTCGTGGACCTCGACTAAGGCAATGTGCTTTCTGGAAGAAGTACCTTCCTCAATTGATGG
CTGCCACGATCGAGAACAGTTCAACCAAAAATTGCACCAACGTTGGAAACCAATTCGTGAGGAATCCAAATTTTTCA
ATACCCACAACACTATTAGTCATACTGGGAATACTGAGTGTCAACTAATGACGCCCTTCCTGATCAAGAGTACCACA
ATTATGTGTTTCGTCACAGTAATTGCTATTAAAATTTATATACTAAATTCAAATGTGAAGTAACGCGATTTACTCA

>PpAChE1-16
MEIRGIVVTTMRPFTGIHSGVDQMFVICLLSLLGVMCQLAEGRHHDLSNTQSFKSGPKHIASVEAAAVSVLGESTLEVSSESDDTI
FTPYLGHGDAVRVVDAELGTLEREGVSAGSDGTSQPRRRNISRRESNPDAEENDPLIVTTDKGKVRGVTLTSPTGKKVDAWMGIPY
AQPPVGALRFRHPRPAERWSGILNATTPPNTCVQIVDTLFGDFPGATMWNPNTNLTEDCLYINVAVPHPRPKNSPVMLWIFGGGFY
SGTSTLDVYDHRTLVAEENIILVSMQYRVASLGFLYLGTPDAPGNAGLFDQHLALRWVRNNIHRFGGDPTRVTLFGESAGAVSVSM
HLLSSLSHDLFQRAILQSGSPTAPWALITRDEAINRTLRLAEAVECPHNRDELSEVLECLRSRDAKQLVNNEWNNLGICEFPFVPV
VDGSFLDESPQRAMATGRFEKTDILTGSNTEEGYYFIIYYLTELLRKEEGITVTREEFLKAVRELNPYVNGAVRQAIVFEYTDWTD
PDNAHSNRDALDKMVGDYHFTCNVNEFAHRYAEEGNNVYMYLYTHRTKANPWPRWTGVMHGDEINYVFGEPLNPSLTYTDEEKEFS
RRIMRYWVNFAKTGNPNPGFVSNLPDWPKHTAHGRQYMELGLNTTYLGRGPRLRQCAFWKKYLPQLMAATIENSSTKNCTNVGNQF
VRNPNFSIPTTLLVILGILSVN*
