# Supplementary material for: Acetylcholinesterase of the sand fly, Phlebotomus papatasi (Scopoli): cDNA sequence, baculovirus expression, and biochemical properties
Source: Parasit Vectors. 2013 Feb 4;6:31. doi: 10.1186/1756-3305-6-31 (PMC3598880; doi:10.1186/1756-3305-6-31)
Supplement: Additional file 2: Figure S2 — Clustal W 2.1 multiple sequence alignment of AChE protein sequences for Torpedo californica [GenBank: 1EA5_A], Phlebotomus papatasi (rPpAChE-16), and Drosophila melanogaster AChE [GenBank: 1QO9_A]. The consensus line below the aligned sequences indicates positions of conserved amino acid identity (*) or similarity (: or.). Positions of the 3 disulfide bond linkages are indicated by numbers above participating cysteine pairs. The members of the catalytic triad (S, E, H) which make up the catalytic site are indicated by (‡) above the participating amino acid. Positions lining the catalytic gorge are indicated by (▼) above the participating amino acid [32,33]. [file 1756-3305-6-31-S2.pdf]

Supplemental Fig. 2

```
T. californica  ------------------------------------------------------------
P. papatasi     MEIRGIVVTTMRPFTGIHSGVDQMFVICLLSLLGVMCQLAEGRHHDLSNTQSFKSGPKHI 60
D. melanogaster ------------------------------------------------------------


T. californica  ------------------------------------------------------------
P. papatasi     ASVEAAAVSVLGESTLEVSSESDDTIFTPYLGHGDAVRVVDAELGTLEREGVSAGSDGTS 120
D. melanogaster ------------------------------------------------------------


T. californica  ----------------DDHSELLVNTKSGKVMGTRVPVLS-SHISAFLGIPFAEPPVGNM 43
P. papatasi     QPRRRNISRRESNPDAEENDPLIVTTDKGKVRGVTLTSPTGKKVDAWMGIPYAQPPVGAL 180
D. melanogaster -----------------VIDRLVVQTSSGPVRGRSVTVQG-REVHVYTGIPYAKPPVEDL 42
                                   . *:* *..* * *  :.     .: .: ***:*:***  :

                                       1  ▼           ▼         1
T. californica  RFRRPEPKKPWSGVWNASTYPNNCQQYVDEQFPGFSGSEMWNPNREMSEDCLYLNIWVPS 103
P. papatasi     RFRHPRPAERWSGILNATTPPNTCVQIVDTLFGDFPGATMWNPNTNLTEDCLYINVAVPH 240
D. melanogaster RFRKPVPAEPWHGVLDATGLSATCVQERYEYFPGFSGEEIWNPNTNVSEDCLYINVWAPA 102
                ***:* *  :  *  *: :*:   .  .* *      *  .*.*  :**** :::*****:*: .*

                                                           ▼      ▼        ▼
T. californica  PR-----------------------------PKSTT----VMVWIYGGGFYSGSSTLDVY 130
P. papatasi     PR-----------------------------PKNSP----VMLWIFGGGFYSGTSTLDVY 267
D. melanogaster KARLRHGRGANGGEHPNGKQADTDHLIHNGNPQNTTNGLPILIWIYGGGFMTGSATLDIY 162
                                               *:.:.    :::**:**** :*::***:*


T. californica  NGKYLAYTEEVVLVSLSYRVGAFGFLALHG------SQEAPGNVGLLDQRMALQWVHDNI 184
P. papatasi     DHRTLVAEENIILVSMQYRVASLGFLYLG-------TPDAPGNAGLFDQHLALRWVRNNI 320
D. melanogaster NADIMAAVGNVIVASFQYRVGAFGFLHLAPEMPSEFAEEAPGNVGLWDQALAIRWLKDNA 222
                :   :.   ::::.*:.***.::*** *         : :****.** ** :*::*:::*

                               ‡                                ▼
T. californica  QFFGGDPKTVTIFGESAGGASVGMHILSPGSRDLFRRAILQSGSPNCPWASVSVAEGRRR 244
P. papatasi     HRFGGDPTRVTLFGESAGAVSVSMHLLSSLSHDLFQRAILQSGSPTAPWALITRDEAINR 380
D. melanogaster HAFGGNPEWMTLFGESAGSSSVNAQLMSPVTRGLVKRGMMQSGTMNAPWSHMTSEKAVEI 282
                : ***:*  :*:******. **. :::*. ::.*.:*.::***: ..**: ::  :. .

                         2              2             ▼        ▼ ▼
T. californica  AVELGRNLNCNLNSDE----ELIHCLREKKPQELIDVEWNVLPFDSIFRFSFVPVIDGEF 300
P. papatasi     TLRLAEAVECPHNRDEL--SEVLECLRSRDAKQLVNNEWNNL---GICEFPFVPVVDGSF 435
D. melanogaster GKALINDCNCNASMLKTNPAHVMSCMRSVDAKTISVQQWNSYS--GILSFPSAPTIDGAF 340
                   * .  :*  .  :    .:: *:*. ..: :    :**      .*  *. .*.:** *

                                      ‡  ▼▼  ▼
T. californica  FPTSLESMLNSGNFKKTQILLGVNKDEGSFFLLYGAPGFSKDSE-SKISREDFMSGVKLS 359
P. papatasi     LDESPQRAMATGRFEKTDILTGSNTEEGYYFIIYYLTELLRKEEGITVTREEFLKAVREL 495
D. melanogaster LPADPMTLMKTADLKDYDILMGNVRDEGTYFLLYDFIDYFDKDDATALPRDKYLEIMNNI 400
                :   .     : :. ::. :** *    :** :*::*        ..:    :.*:.::. :.

                                                          3
T. californica  VPHANDLGLDAVTLQYTDWMDDNNGIKNRDGLDDIVGDHNVICPLMHFVNKYTKFGNGTY 419
P. papatasi     NPYVNGAVRQAIVFEYTDWTDPDNAHSNRDALDKMVGDYHFTCNVNEFAHRYAEEGNNVY 555
D. melanogaster FGKATQAEREAIIFQYTSWEG-NPGYQNQQQIGRAVGDHFFTCPTNEYAQALAERGASVH 459
                   ..     :*: ::**.*  .  :  .  .*:: :.   ***:  .  *     .:.:   :: *  ..:

                            ▼       ‡ ▼
T. californica  LYFFNHRASNLVWPEWMGVIHGYEIEFVFGLPLVKELNYTAEEEALSRRIMHYWATFAKT 479
P. papatasi     MYLYTHRTKANPWPRWTGVMHGDEINYVFGEPLNPSLTYTDEEKEFSRRIMRYWVNFAKT 615
D. melanogaster YYYFTHRTSTSLWGEWMGVLHGDEIEYFFGQPLNNSLQYRPVERELGKRMLSAVIEFAKT 519
                 * :.**:.    *  .* **:** **::.** **   .* *    *. :.:*::      ****

                                                            3
T. californica  GNPNE-PHSQESKWPLFTTKEQKFIDLNTEP--MKVHQRLRVQMCVFWNQFLPKLLNATA 536
P. papatasi     GNPNPGFVSNLPDWPKHTAHGRQYMELGLNT--TYLGRGPRLRQCAFWKKYLPQLMAATI 673
D. melanogaster GNPAQ----DGEEWPNFSKEDPVYYIFSTDDKIEKLARGPLAARCSFWNDYLPKVRSWAG 575
                ***       :  .**  .: .    :   :. :       : :       * **:.:**::    :

T. californica  C------------------------------------ 537
P. papatasi     ENSSTKNCTNVGNQFVRNPNFSIPTTLLVILGILSVN 710
D. melanogaster TCDGDSGSAS--------------------------- 585
```
